# The impact of the COVID-19 pandemic on non-COVID induced sepsis survival

**DOI:** 10.1186/s12871-021-01547-8

**Published:** 2022-01-05

**Authors:** Matthias Unterberg, Tim Rahmel, Katharina Rump, Alexander Wolf, Helge Haberl, Alexander von Busch, Lars Bergmann, Thilo Bracht, Alexander Zarbock, Stefan Felix Ehrentraut, Christian Putensen, Frank Wappler, Thomas Köhler, Björn Ellger, Nina Babel, Ulrich Frey, Martin Eisenacher, Daniel Kleefisch, Katrin Marcus, Barbara Sitek, Michael Adamzik, Björn Koos, Hartmuth Nowak, on behalf of the SepsisDataNet.NRW research group, Michael Adamzik, Moritz Anft, Thorsten Annecke, Nina Babel, Maha Bazzi, Lars Bergmann, Christian Bode, Thilo Bracht, Alexander von Busch, Jerome M. Defosse, Stefan F. Ehrentraut, Martin Eisennacher, Björn Ellger, Christian Ertmer, Ulrich H. Frey, Katrin Fuchs, Helge Haberl, Dietrich Henzler, Daniel Kleefisch, Thomas Köhler, Björn Koos, Ulrich Limper, Katrin Marcus, Hartmuth Nowak, Daniel Oswald, Christian Putensen, Tim Rahmel, Katharina Rump, Jens-Christian Schewe, Elke Schwier, Barbara Sitek, Matthias Unterberg, Frank Wappler, Katrin Willemsen, Alexander Wolf, Alexander Zarbock, Birgit Zuelch

**Affiliations:** 1 Klinik für Anästhesiologie, Intensivmedizin und Schmerztherapie, Universitätsklinikum Knappschaftskrankenhaus Bochum, Bochum, Germany; 2 Ruhr University Bochum, Medical Faculty, Medizinisches Proteom-Center, and Center for Protein Diagnostics (ProDi), Medical Proteome Analysis, Ruhr University Bochum, 44801 Bochum, Germany; 3 Klinik für Anästhesiologie, Operative Intensivmedizin und Schmerztherapie, Universitätsklinikum Münster, Münster, Germany; 4 Klinik für Anästhesiologie und Operative Intensivmedizin, Universitätsklinikum Bonn, Bonn, Germany; 5 Department of Anaesthesiology and Operative Intensive Care Medicine, University of Witten/Herdecke, Cologne Merheim Medical School, Cologne, Germany; 6 Department of Anesthesiology, Surgical Intensive Care, Emergency and Pain Medicine, Ruhr-University Bochum, Klinikum Herford, Herford, Germany; 7 Klinik für Anästhesiologie, Intensivmedizin und Schmerztherapie, Klinikum Westfalen, Dortmund, Germany; 8 Department for Anesthesiology, Intensive Care Medicine and Pain Therapy, Klinikum Westfalen, Dortmund, Germany; 9 Center for Translational Medicine, Medical Clinic I, Marien Hospital Herne, University Hospital of the Ruhr-University Bochum, Herne, Germany; 10 Klinik für Anästhesiologie, Operative Intensivmedizin, Schmerz- und Palliativmedizin, Marien Hospital Herne, Universitätsklinikum der Ruhr-Universität Bochum, Bochum, Germany

**Keywords:** COVID-19 pandemic, Sepsis, 30-day mortality

## Abstract

**Background:**

The COVID-19 pandemic has taken a toll on health care systems worldwide, which has led to increased mortality of different diseases like myocardial infarction. This is most likely due to three factors. First, an increased workload per nurse ratio, a factor associated with mortality. Second, patients presenting with COVID-19-like symptoms are isolated, which also decreases survival in cases of emergency. And third, patients hesitate to see a doctor or present themselves at a hospital. To assess if this is also true for sepsis patients, we asked whether non-COVID-19 sepsis patients had an increased 30-day mortality during the COVID-19 pandemic.

**Methods:**

This is a post hoc analysis of the SepsisDataNet.NRW study, a multicentric, prospective study that includes septic patients fulfilling the SEPSIS-3 criteria. Within this study, we compared the 30-day mortality and disease severity of patients recruited pre-pandemic (recruited from March 2018 until February 2020) with non-COVID-19 septic patients recruited during the pandemic (recruited from March 2020 till December 2020).

**Results:**

Comparing septic patients recruited before the pandemic to those recruited during the pandemic, we found an increased raw 30-day mortality in sepsis-patients recruited during the pandemic (33% vs. 52%, *p* = 0.004). We also found a significant difference in the severity of disease at recruitment (SOFA score pre-pandemic: 8 (5 - 11) vs. pandemic: 10 (8 - 13); *p* < 0.001). When adjusted for this, the 30-day mortality rates were not significantly different between the two groups (52% vs. 52% pre-pandemic and pandemic, *p* = 0.798).

**Conclusions:**

This led us to believe that the higher mortality of non-COVID19 sepsis patients during the pandemic might be attributed to a more severe septic disease at the time of recruitment. We note that patients may experience a delayed admission, as indicated by elevated SOFA scores. This could explain the higher mortality during the pandemic and we found no evidence for a diminished quality of care for critically ill sepsis patients in German intensive care units.

## Background

Since December 2019, the ongoing pandemic of coronavirus disease 2019 (COVID-19) has posed significant challenges to healthcare systems worldwide [1]. However, the German healthcare system seemed relatively well equipped with approximately 28,000 ICU beds (22,000 with the option for mechanical ventilation) for 83 million inhabitants. Thus, while lacking ICU capacity did not affect Germany as much as it did other countries, three main factors impacted critically ill patients in Germany during the pandemic. First, a shortage of intensive care nurses and the increased workload during the pandemic led to an increased patient-to-nurse ratio, which is associated with a higher mortality for critically ill patients [2]. Secondly, all COVID-19 patients were at least initially handled under isolation conditions, to avoid SARS-CoV-2 outbreaks in the hospitals. This was also true for patients presenting with COVID-19 like symptoms until a PCR test could rule out a SARS-CoV-2 infection, although isolation is well described to be associated with an increased mortality for critically ill patients [3, 4]. And third, a general anxiety in the population to contract COVID-19 [5] might lead to a delayed ICU admission, which would mean more severe disease at admission. Disease severity (i.e., admission SOFA score) is also strongly associated with worse outcome in septic patients. This hesitancy of patients to see a physician or to be admitted to the hospital during the pandemic has been described for nonemergency conditions such as elective surgery [6], but also for more severe conditions such as cancer [7] despite the expectable negative effect on the clinical course. Even patient admission for emergency conditions such as myocardial infarction and stroke decreased during the pandemic [6]. All three factors could influence the mortality of critically ill patients independent of a COVID-19 diagnosis. In line with this, the mortality of patients suffering from severe COVID-19 is surprisingly high [8, 9], when compared to other septic patients. Hence, it was reasonable to assume that the ongoing COVID-19 pandemic had also a negative impact on the prognosis of critically ill non-COVID-19 patients. Nonetheless, the consequences of the pandemic and its subsequent factors for the clinical outcomes in these patients are currently unknown. Therefore, we hypothesized that non-COVID-19 septic patients would have (1) a higher 30-day mortality and (2) a more severe disease at admission. To address this, we performed a post hoc analysis of the prospective, multi-center observational study SepsisDataNet.NRW.

## Methods

### Study design and cohort

The SepsisDataNet.NRW study (German Clinical Trial Registry No. DRKS00018871; http://www.sepsisdatanet.nrw) prospectively enrolled patients fulfilling SEPSIS-3 criteria in a multicentric approach. The study was approved by the Ethics Committee of the Medical Faculty of Ruhr-University Bochum (Registration No. 5047–14) or the responsible ethics committee of each respective study center. Patients were recruited after written informed consent over a period from 1st of March 2018 until 31st of December of 2020 at seven different ICUs of tertiary care and university hospitals in the German state of North Rhine-Westphalia. Eligible for inclusion were adult patients with a sepsis diagnosis within the previous 36 h according to the current SEPSIS-3 definition (suspected/proven infection and an increase of Sequential Organ Failure Assessment (SOFA) score by two points or more).

Exclusion criteria were as follows:

1. Age below 18 years at the time of ICU admission
2. Withdrawal or withhold of consent
3. Withdrawal of treatment

**Fig. 1:**
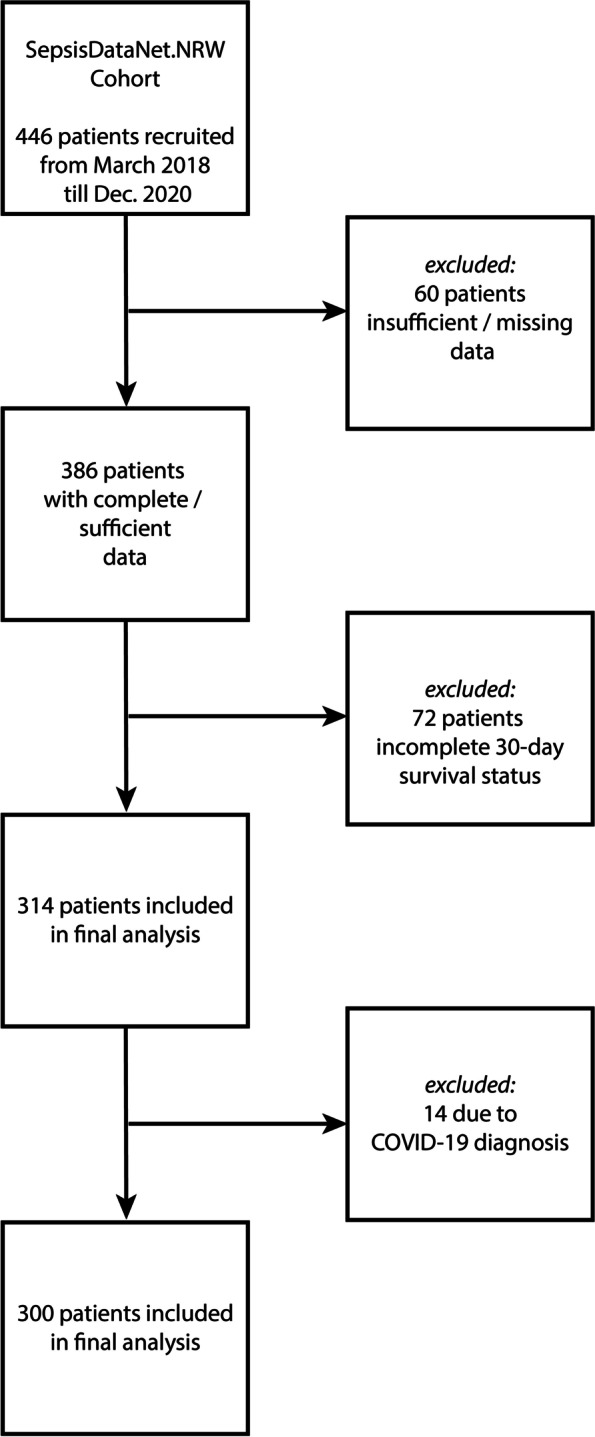
Flow chart for identification of the cohort for the final analysis. Four hundred forty-six septic patients were recruited to the SepsisDataNet.NRW study by December 2020. Of these 60 patients were excluded due to missing data. Of the remaining 386 patients, 72 were excluded due to incomplete 30-day survival status. Another 14 patients were excluded from analysis because of a COVID-19 diagnosis and the remaining 300 patients were included in the final analysis

For the cohort reported in this study, we considered sepsis patients, negative for SARS-CoV-2, who were recruited between 1st of March 2018 and 31st of December 2020, with complete information of 30-day mortality status and clinical dataset. Septic patients positive for SARS-CoV-2, who were also enrolled in this study, were excluded from this analysis (Fig. 1).

### Definition of groups and endpoints

Critically ill non-COVID-19 patients recruited from 1st of March 2018 till 29th of February 2020 were defined as the pre-pandemic subgroup. Patients recruited from the 1st of March 2020 till 31st of December of 2020 were defined as the pandemic subgroup. To assess the impact of COVID-19 measures on the outcome of critically ill non-COVID-19 patients, we compared the following clinical endpoints between the two groups: 30-day mortality, initial SOFA score, ICU length of stay and rate of mechanical ventilation.

### Calculation of the SOFA score

Recorded clinical data were used to calculate the SOFA score and its respective components (CNS, renal, liver, cardiovascular, respiratory and coagulation) at the time of study inclusion. We strictly followed the general guidelines and proposals for calculating the SOFA score as described by Lambden et al. [10] For sedated patients, the last pre-intubation Glasgow Coma Scale (GCS) score was estimated and carried forward throughout sedation. If GCS prior to sedation was unknown, a value of 15/15 was assumed. Missing data to calculate the initial SOFA-score were completed by including data from ±12 h of the onset of sepsis. Additionally, the SOFA score was validated by a physician at each study site.

### Calculation of the Simplified Acute Physiology Score (SAPS-II) without GCS

Recorded clinical data, age and the past medical history were used to calculate the SAPS II score at admission to the ICU. We strictly followed the guidelines for calculating the SAPS II score using the worst values over the first 24 h after admission. In addition, the Glasgow Coma Scale (GCS) was not assessed, as is common in German ICU-scoring. Whenever missing data at day one prevented the calculation, data of day two was used. Either the SAPS II score was calculated by a physician at each study site or, if former calculated for hospital billing purposes, plausibility was rechecked by a physician at each study site.

### Focus of infection

The clinical focus of infection and sepsis was recorded by a post hoc revision of patients’ ICU discharge reports by a physician at each study site. If this was inconclusive, the detailed medical record of the ICU-stay was additionally considered. In some cases, more than one suspected site of infection remained after data revision. In these cases, the most likely underlying focus was determined after conference with a second physician. For classification the results were categorized according to organ-systems which are cardiovascular, central nervous system, genitourinary, intraabdominal, lower respiratory tract, musculoskeletal and skin & soft tissue.

### Cytokine concentration in serum

As part of the SepsisDataNet.NRW study biomaterials (e.g., serum) were collected at day 1 after recruitment (less than 36 h after initial diagnosis). To assess the immune reaction, these serum samples were used to quantify the concentration of cytokines at the time of recruitment. The LegendPlex Human Inflammation Panel 1 (Cat No. 740809, Biolegend, San Diego) was used according to manufacturer’s instructions. The panel measures the concentration of 13 cytokines (i.e., IL1B, IFNa2, IFNg, TNFa, MCP1, IL6, IL8, IL10, IL12, IL17a, IL18, IL23 and IL33). Briefly, 25 uL serum samples were mixed with assay buffer and incubated with LegendPlex beads for antigen capture (2 h). The plate was spun down, and the beads were subsequently washed and incubated with detection antibodies for 1 h. After adding the Fluorophore and further incubation, the beads were washed thrice. Finally, the beads were resuspended, and fluorescence was measured in a flow cytometer (Canto II, BD Biosciences, CA). The cytokine concentration was quantified using a standard curve with a standard supplied by the manufacturer. Data analysis was performed using the LEGENDplex software (BioLegend, San Diego) provided with the kit. When the recorded concentration of a cytokine was below the lower limit of detection (LOD) the value was treated as 0 ng/mL, additionally if a value was recorded as higher than the upper LOD it was treated as the upper LOD.

### Propensity score matching

The 42 pandemic patients were matched to 42 pre-pandemic patients using a propensity score with the variables: initial SOFA score, age, and gender.

### Statistics

Statistical analysis and propensity score matching were performed using the IBM® SPSS Statistics software version 27. The 30-day survival was compared between the groups using a Kaplan Meier analysis and the log-rank test. Independence of risk factors was evaluated using a Cox Regression analysis. The Cox Regression analysis was done using the variables pandemic, age, SAPS-II Score at admission, SOFA score at recruitment and cytokine concentrations of IL1B, IFNa2, IFNg, TNFa, MCP1, IL6, IL8, IL10, IL12, IL17a, IL18, IL23 and IL33 at the day of recruitment. The cohorts were compared for demographics and descriptive data using a nonparametric Mann Whitney U test. Categorical variables (except for 30-day survival) were compared using the Fisher’s Exact test. A *p*-value of lower than 5% was considered significant. If not stated otherwise, the data is always depicted as mean ± standard deviation (SD).

## Results

### Cohort description

**Fig. 2:**
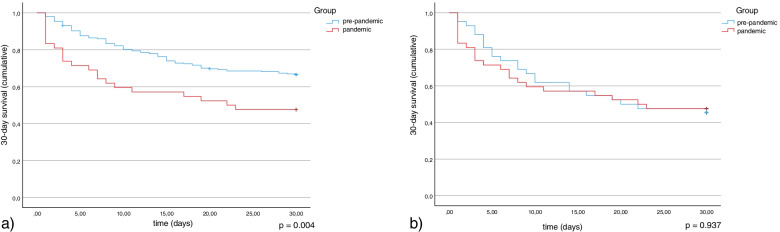
Kaplan Meier analysis considering the 30-day survival of sepsis patients recruited before March 2020 (pre-pandemic, blue) and after March 2020 (pandemic, red). **A**: In an unbiased analysis a clear difference between the groups can be seen (*p* = 0.004). **B**: When adjusted for the higher SOFA score during the pandemic, there is no significant difference between the two groups (*p* = 0.798)

Three hundred patients were included in the final analysis (Fig. 1). They were separated into the pre-pandemic (*n* = 258) and pandemic (*n* = 42) groups. The overall 30-day mortality in the pre-pandemic group was 33%. In the pandemic group, the rate was significantly higher with 52% (*p* = 0.004; Fig. 2a, Table 1). Demographic parameters like sex (62% male vs 62% male pre-pandemic and pandemic respectively; *p* = 1.000) and age (64 ± 14 years vs. 65 ± 16 years pre-pandemic and pandemic, respectively; *p* = 0.627) did not significantly differ (Table 1). Also, the focus of the underlying infection did not change significantly during the pandemic (*p* = 0.522 over all groups). Furthermore, ICU length of stay (median: 7.7 days; IQR: 1.7–13.7 vs 10.8 days; IQR: 0.3–21-3 pre-pandemic and pandemic respectively; *p* = 0.443) and percentage of patients in need of mechanical ventilation (86% vs 97% pre-pandemic and pandemic respectively; *p* = 0.081) were not significantly different before and after the onset of the COVID-19 pandemic. However, the median SOFA-score at the day of enrollment was significantly higher after March 2020 than before (8, IQR: 5–11 vs. 10, IQR: 7.5–12.5 pre-pandemic and pandemic respectively; *p* < 0.001). In addition, the median Simplified Acute Physiology Score (SAPS-II) at ICU admission was not significantly higher during the pandemic (38, IQR: 28–48 vs. 39, IQR: 28–50 pre-pandemic and pandemic respectively; *p* < 0.242). On the molecular level, the two cohorts differed only in the serum concentration at admission of interleukin (IL)-1β (9 ± 25 pg/mL vs. 12 ± 13 pg/mL pre-pandemic and pandemic respectively; *p* = 0.022) and – most notably – TNF-α (18 ± 100 pg/mL vs. 4 ± 5 pg/mL pre-pandemic and pandemic respectively; *p* = 0.003).

**Table 1:** Base characteristics of the patient cohort recruited pre-pandemic vs. the patient cohort recruited during the pandemic. A value for *p* < 0.05 was considered statistically significant

	Pre-Pandemic	Pandemic	***p***-value
**n**	258	42	
**Male gender n (%)**	159 (62%)	26 (62%)	*p* = 1.000
**Age mean in years (+/− SD)**	64 (14)	65 (16)	*p* = 0.627
**Initial SOFA score (median [IQR])**	8 [5–11]	10 [8–13]	***p*** **< 0.001**
**SAPS-II at ICU admission (median [IQR]**	38 [28–48]	39 [28–50]	*p* = 0.242
**Mechanical ventilation n/m (%)**	175/203 (86%)	32/33 (97%)	*p* = 0.081
**Focus of infection n/m (%)**			
**- Central Nervous System**	3/215 (1%)	2/33 (6%)	*p* = 0.076
**- Lower Respiratory Tract**	101/215 (47%)	15/33 (45%)	*p* = 0.684
**- Skin & Soft Tissue**	14/215 (7%)	0/33 (0%)	n.a.
**- Genitourinary**	17/215 (8%)	2/33 (6%)	*p* = 0.710
**- Cardiovascular**	11/215 (5%)	2/33 (6%)	*p* = 0.821
**- Intra-abdominal**	58/215 (27%)	10/33 (30%)	*p* = 0.690
**- Musculoscetal**	11/215 (5%)	2/33 (6%)	*p* = 0.821
**ICU length of stay, days (median [IQR])**	7.7 [1.7–13.7]	10.8 [0.3–21.3]	*p* = 0.433
**30-day mortality n (%)**	86 (33%)	22 (52%)	***p*** **= 0.004**
**Cytokine Concentration mean in pg/mL in Serum at enrollment (+/− SD)**			
**TNF-α**	18 (100)	4 (5)	***p*** **= 0.003**
**IL-6**	958 (2078)	2015 (2987)	*p* = 0.225
**IL-10**	69 (237)	62 (225)	*p* = 0.056
**IL-1b**	4 (25)	12 (13)	***p*** **= 0.022**

To adjust for the more severe septic disease state at the time of diagnosis, we matched the pandemic sepsis patients with pre-pandemic patients using a propensity score matching approach. In this matched pre-pandemic cohort, mortality was not significantly different from the mortality in the pandemic cohort (55% vs. 52% pre-pandemic and pandemic respectively; *p* = 0.937, Table 2, Fig. 2b) suggesting the observed survival decrease for sepsis patients during the pandemic might be due to a higher disease severity and / or older age. This is supported by a Cox-Regression analysis identifying the factor “pandemic” as non-independent (*p* = 0.904), with SOFA score at admission (*p* < 0.001) and IL-10 concentration in serum (*p* = 0.008) being the only independent risk factors.

**Table 2:** Base characteristics of the matched pairs cohorts. For each patient recruited during the pandemic a propensity score matched patient recruited in the pre-pandemic period was selectd. A value for *p* < 0.05 was considered statistically significant

	Pre-Pandemic	Pandemic	***p***-value
**n**	42	42	
**Male gender n (%)**	28 (67%)	26 (62%)	*p* = 0.651
**Age mean in years (+/− SD)**	66 (13)	65 (16)	*p* = 0.883
**Initial SOFA score [IQR]**	11 [8–14]	11 [10–12]	*p* = 0.943
**SAPS-II at ICU admission (median [IQR]**	40 [33–47]	39 [28–50]	*p* = 0.766
**Mechanical ventilation n (%)**	34/35 (97%)	40/42 (95%)	*p* = 0.667
**Focus of infection n (%)**			
**Central Nervous System**	1/38 (3%)	2/33 (6%)	*p* = 0.473
**Lower Respiratory Tract**	20/38 (53%)	15/33 (45%)	*p* = 0.546
**Skin & Soft Tissue**	2/38 (5%)	0/33 (0%)	n.a.
**Genitourinary**	3/38 (8%)	2/33 (6%)	*p* = 0.763
**Cardiovascular**	2/38 (5%)	2/33 (6%)	*p* = 0.884
**Intra-abdominal**	8/38 (21%)	10/33 (30%)	*p* = 0.373
**Musculoscetal**	2/38 (5%)	2/33 (6%)	p = 0.884
**ICU length of stay, days (median [IQR])**	9.0 [1.9–16.1]	10.8 [0.3–21.3]	*p* = 0.773
**30-day mortality n (%)**	23 (55%)	22 (52%)	p = 0.937
**Cytokine Concentration mean in pg/mL in Serum at enrollment (+/− SD)**			
**TNF-α**	32 (144)	4 (5)	***p*** **< 0.001**
**IL-6**	1758 (3194)	2015 (2987)	*p* = 0.849
**IL-10**	115 (257)	62 (225)	*p* = 0.663
**IL-1b**	17 (58)	12 (13)	*p* = 0.085

Cytokine concentrations were compared for the matched cohort as well (Table 2). In the matched analysis IL-1b (17 ± 58 pg/ml vs 12 ± 13 pg/ml pre-pandemic and pandemic, respectively; *p* = 0.085) is no longer significantly different between the groups. Strikingly, the TNF-α levels were still significantly higher at admission in the pre-pandemic group compared to non-COVID-19 sepsis patients during the pandemic (32 ± 144 pg/ml vs 4 ± 5 pg/ml pre-pandemic and pandemic respectively; *p* < 0.001).

## Discussion

In this retrospective analysis of a prospectively recruited, multicentric study, we find a higher raw mortality of sepsis patients during the COVID-19 pandemic, as compared to the pre-pandemic period, a finding that is mirrored by a recently published study by Bodilsen et al. [11]. In their study the authors could not evaluate whether the increased mortality in sepsis was due to an overburdened healthcare system or patients not seeking care when needed. Our data indicate that the quality of care in the ICUs did not suffer significantly as a propensity score adjusted analysis showed that the SOFA score and age adjusted patient outcome did not significantly decrease during the pandemic. Given the higher patient-to-nurse ratio, the decreased time spent per patient, and the initial isolation procedures, this might be surprising. We can speculate that the initial isolation until a PCR test could rule out a SARS-CoV-2 infection might not be sufficient to affect survival, as work regarding isolation procedures mainly focusses on groups with longer isolation times [12]. In addition, we can speculate that the usual indicators such as patient-to-nurse ratio, or workload-per-nurse ratio do not apply during the pandemic as health care workers go beyond their limits to provide care for their patients as can be seen by the high number of burnout syndromes in nurses and other health professionals [13].

As our data point towards patients not seeking care when needed, rather than a degradation of quality of care, we assumed that sepsis patients during the pandemic would present with a more severe disease state than before. This assumption is supported by our data as patients recruited during the pandemic suffered from more and stronger organ dysfunction (as indicated by the higher SOFA score at recruitment) than before the pandemic. One explanation for this could be, that generally sicker people (e.g., those with pre-existing conditions such as cancer) were admitted to the ICUs and then recruited to our study. In order to evaluate this, we assessed the Simplified Acute Physiology Score (SAPS II) [14] at ICU admission. The SAPS II score records the general physical state of the patient, including age and chronic health conditions and was specifically designed to compare study cohorts [14]. The SAPS II score did not differ significantly between our two groups, which supports the notion that, while these patients have a worse stage of septic disease with more severe organ dysfunction (SOFA score), their general physical state of health (including pre-existing conditions) did not differ.

In addition to the higher SOFA score, we also found higher levels of TNF-α in the serum of patients recruited before the pandemic than in those patients admitted after March 2020. The exact dynamics of TNF-α during sepsis is still controversial, but there is some work suggesting TNF-α as an early, proinflammatory cytokine [15, 16]. As this could be affected by the focus of the initial infection, we evaluated this and found that the general makeup of the infection foci did not change during the pandemic. While we cannot exclude other confounding factors, this would support the notion that sepsis patients presented earlier at ICUs before the COVID-19 pandemic. Further work is needed in order validate this hypothesis.

It has been reported that during the pandemic, patients suffered from a hesitancy to see a physician or be admitted to the hospital for fear of contracting COVID-19 [5], which could explain a later admission to the ICU. While this might be surprising for a critical illness such as sepsis, the effect of delayed or avoided hospital admission during the COVID-19 pandemic is expectable for medical conditions without serious consequences and could be shown in a very high extent for elective surgery like knee arthroplasty [6]. Furthermore, patients suffering from malignant diseases (i.e., cancer) also suffered from delayed therapy onset [7] despite an expectable negative effect on the clinical course. Even patient admission for emergency conditions like myocardial infarction and stroke was decreased during the pandemic [6]. This is also unexpected, as the likelihood of a negative outcome is significantly increased because of delayed diagnosis and therapy in these cases. Additionally, hospital admissions for sepsis decreased during lockdown phases as shown by Bodilsen et al. [11]. This, taken together with our data, suggests that patients suffering from severe infections and circumstances that have to be considered as pre-septic or early septic conditions could also be affected by this delay.

### Limitations

An important limitation is a small patient cohort recruited during the pandemic (*n* = 42). That means that the matched analysis could have too small sample size to observe relevant effects. However, post hoc power calculations reveal that we will still be able to observe medium effects (effect size = 0.3) with a power of > 80%. However, larger studies are needed to validate our hypotheses. Furthermore, from a non-significantly different propensity score adjusted mortality, we suggested a stable standard of care during the pandemic. This does not necessarily have to be true as there are other parameters to measure the quality of care that are not considered. In our opinion, however, 30-day mortality is the most relevant and quantifiable endpoint. We can thus not exclude a decrease in the quality of care that does not reach a level that would significantly impact survival.

Another limitation in our cohort is a possible recruitment bias. As in the SepsisDataNet.NRW study, blood samples of all patients are collected at the time of enrollment and further defined points of time, patients are recruited from Sunday Morning till Friday morning, and patients are not included if they are admitted to the ICU on Friday afternoon or Saturday.

A major limitation of this study is the effect of the pandemic itself. Beginning from March 2020, research on non-COVID-19 topic were not prioritized and some of the clinics of our network stopped or slowed either recruitment or data provisioning to our study. This resulted in a recruitment bias where sepsis patients recruited during the pandemic are only provided by two centers, while pre-pandemic patients were recruited in all seven centers.

## Conclusion

Most importantly we found an increased raw 30-day mortality for sepsis patients during the COVID-19 pandemic. However, when adjusted for disease severity we found no significant difference in survival. The higher disease severity might therefore serve as the reason of a higher raw 30-day mortality of sepsis patients recruited between March and December 2020. Taking this into account, we suggest that the quality of care on ICUs did not diminish significantly despite the unique burdens, that the SARS-CoV-2 pandemic put on the German health care system. However, we report a concerningly high initial SOFA score, possibly due to delayed patient admission in sepsis patients during the pandemic. Thus, in line with other emergency patients like those suffering from stroke or myocardial infarction, sepsis patients seem to be put at a disadvantage by pandemic conditions despite a continuously high quality of care.

## Data Availability

All data reported on in this study will be available from the corresponding author upon reasonable request.
